# Chemoresistance Mediated by ceRNA Networks Associated With the PVT1 Locus

**DOI:** 10.3389/fonc.2019.00834

**Published:** 2019-08-27

**Authors:** Olorunseun O. Ogunwobi, Adithya Kumar

**Affiliations:** ^1^Department of Biological Sciences, Hunter College of the City University of New York, New York, NY, United States; ^2^Joan and Sanford I. Weill Department of Medicine, Weill Cornell Medicine, Cornell University, New York, NY, United States

**Keywords:** PVT1, lncRNA, ceRNA, miRNA, chemoresistance, carcinogenesis, 8q24, cancer

## Abstract

Competitive endogenous RNA (ceRNA) networks have emerged as critical regulators of carcinogenesis. Their activity is mediated by various non-coding RNAs (ncRNAs), including long non-coding RNAs and microRNAs, which competitively bind to targets, thereby modulating gene expression and activity of proteins. Of particular interest, ncRNAs encoded by the 8q24 chromosomal region are associated with the development and progression of several human cancers, most prominently lncPVT1. Chemoresistance presents a significant obstacle in the treatment of cancer and is associated with dysregulation of normal cell processes, including abnormal proliferation, differentiation, and epithelial-mesenchymal transition. CeRNA networks have been shown to regulate these processes via both direct sponging/repression and epigenetic mechanisms. Here we present a review of recent literature examining the contribution of ncRNAs encoded by the *PVT1* locus and their associated ceRNA networks to the development of resistance to common chemotherapeutic agents used to treat human cancers.

## Introduction

Competitive endogenous RNA (ceRNA) networks have increasingly been found to play an important role in carcinogenesis (1). These networks are characterized by “sponging” activity, whereby non-coding RNAs (ncRNAs) competitively bind and repress targets, often demonstrating reciprocal activity (2, 3). Long non-coding RNAs (LncRNAs) are generally classified as ncRNA transcripts 200 nucleotides or greater in length and are involved in both transcriptional and post-transcriptional gene regulation, including genome organization (4, 5). Several lncRNAs have been shown to play a role in carcinogenesis, a prominent example being lncPVT1, which is homologous to the mouse plasmacytoma variant translocation 1 gene.

*PVT1* is located downstream of proto-oncogene *MYC* on chromosomal region 8q24, a known cancer susceptibility locus (6). MYC expression has been shown to be highly reliant on *PVT1*—*PVT1* is increased in nearly 98% of cancers displaying overexpression of *MYC* (7). It was recently was found that the *PVT1* promoter can behave as a tumor suppressor DNA boundary element by competing with the *MYC* promoter *in cis* for shared enhancers within the gene locus (8). LncPVT1 can also regulate several downstream components of the MYC pathway (9). Their complex relationship emphasizes the importance of this gene locus to cancer progression.

MicroRNAs (miRNAs) are small ncRNAs, roughly 18–25 nucleotides in length (10). miRNAs can induce translational repression of target mRNA by recruiting the RNA-induced silencing complex (RISC) and binding to miRNA response elements (MREs) (11). LncRNA have been shown to reduce miRNA-mediated translational repression by sequestering miRNAs or competitively binding targets, for example, lncPVT1 has been shown to regulate the activity of its own miRNA transcripts (12, 13). The *PVT1* gene encodes for six microRNAs: miR-1204, miR-1205, miR-1206, miR-1207-3p, miR-1207-5p, and miR-1208 (14, 15). These transcripts have been shown to participate in ceRNA networks in many cancers, exerting both oncogenic and tumor suppressive roles. Circular RNAs (circRNAs) represent another class of ncRNAs that are formed by bonding of the 3′ and 5′ ends of RNA (5).

Chemotherapy resistance presents a significant impediment to successful treatment of most cancers, leading to diminished survival and higher recurrence rates. Cancers can exhibit either primary/intrinsic chemoresistance, for example, via tumor heterogeneity, or secondary/acquired chemoresistance via mechanisms such as target inactivation or alteration, drug efflux, cell death inhibition, DNA damage repair, epigenetics, mutations, or epithelial-mesenchymal transition (EMT) (16).

This paper aims to present a review of primary literature examining the role of lncRNA PVT1, associated miRNA transcripts, and their respective ceRNA networks in the development of resistance to common chemotherapeutic agents used to treat human cancers. This area of research is a promising target for the development of new chemotherapeutics or enhancement of existing treatment regimens.

## Breast Cancer

Breast cancer remains the leading cause of cancer death among women under the age of 60 (17). Triple-negative breast cancers (TNBCs) are among the most difficult to treat and have the worst prognosis among breast cancer subtypes due to the lack of available targeted therapy (18). Taxane-based chemotherapy remains the primary treatment approach for TNBC and metastatic breast cancer. Despite their general success, response rates for paclitaxel and docetaxel are low in many subtypes (19, 20).

MiR-1207 has been found to be elevated in several cancers, including young breast cancer patients (21). Leucine zipper tumor suppressor gene 1 protein (LZTS1), a tumor suppressor, was found to be downregulated in paclitaxel-resistant breast cancer (22). MiR-1207-5p can promote chemoresistance in TNBC cells by inhibiting LZTS1, thereby decreasing cell growth arrest and apoptosis in response to paclitaxel (23). Furthermore, there was downregulation of *Bax* and upregulation of *Bcl-2*, pro-apoptotic and anti-apoptotic genes, respectively.

Although there is evidence for a regulatory role for miR-1207-5p in TNBC, several questions remain. *LZST1* was chosen due to its role as a tumor suppressor, but other apoptotic pathways may also be targeted by miR-1207-5p. Additionally, alternate mechanisms may contribute to overall drug resistance that were not studied here, including proliferative and drug efflux pathways, which are known to be active in breast cancer (24, 25).

MiR-1207-5p has also been found to negatively regulate transcription factor STAT6 in invasive breast cancer, leading to disinhibition of CDKN1A/B, increased proliferation, and cell cycle progression (26). MiR-1204 has been shown to promote tumorigenesis, EMT, and metastasis in breast cancer by targeting the *vitamin D receptor* gene (VDR) (27). The role of vitamin D signaling in cancer is well-studied; calcitriol, the active metabolite of vitamin D, can improve chemosensitivity in breast cancer (28–31). Ablation of VDR has also been shown to promote breast tumorigenesis in mice (32, 33). The aforementioned studies are promising, and future research should further explore possible ceRNA networks involving miR-1204 and miR-1207 in breast cancer.

## Cervical Cancer

Cervical cancer (CC) is the second most common cancer among women aged 20–39 years in the United States (17). HPV infection has been shown to contribute to its development and coincide with 99.7% of cases (34). Two viral HPV oncoproteins, E6 and E7, have been implicated in tumorigenesis via degradation of tumor suppressor p53 and interaction with pRb, respectively (35). Paclitaxel-based chemotherapy is commonly used in conjunction with cisplatin as first-line chemotherapy for CC. (36).

LncPVT1 can regulate miR-195 via both epigenetic and sponging mechanisms, thereby disinhibiting downstream activation of SMAD3, promoting EMT, and inducing paclitaxel resistance in CC (37). The MiR-195 was previously been shown to behave as a tumor suppressor in CC by repressing SMAD3, a member of the SMAD family of transcription factors which mediate the TGF-β family of cytokines, responsible for cell proliferation and differentiation (38, 39). MiR-195 and lncPVT1 have reciprocal sponging activity, whereby miR-195 overexpression reduces expression of lncPVT1 and PVT1 knockdown increases miR-195 expression (37). Overexpression of miR-195 or PVT1 knockdown resulted in downregulation of mesenchymal markers (vimentin, fibronectin) and upregulation of epithelial markers (E-cadherin) in response to paclitaxel treatment.

**Figure 1:**
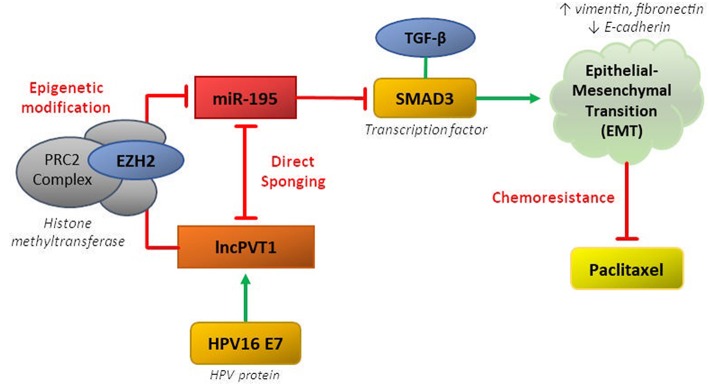
CeRNA networks involving lncPVT1 and its associated transcripts are generally mediated by two mechanisms: (1) direct sponging of other RNA transcripts/proteins or (2) epigenetic modification. Both of these mechanisms have been shown to be active in regulating chemoresistance in cervical cancer.

LncPVT1 can also epigenetically regulate miR-195 by inducing H3K27me3 methylation in the *miR-195* promoter region via recruitment of EZH2. LncPVT1 was also shown recruit EZH2 in lung, hepatocellular, and thyroid cancers (40–42). Additionally, knockdown of HPV16 E7 decreased levels of lncPVT1 and increased levels of miR-195. This ceRNA network involving lncPVT1 and miR-195 in cervical cancer characterizes the typical complex pathway mediating chemoresistance in many cancers (Figure 1).

Previous studies have shown related roles for lncPVT1 in CC. It can sponge miR-424 to promote cell proliferation, invasion and migration, as well as regulate expression of miR-200b by recruiting EZH2 (43, 44). LncRNA HOTAIR has also been shown to interact with HPV16 E7 to potentially contribute to cervical carcinogenesis (45). Approximately 14 other lncRNAs have been shown to be involved in CC and there is significant research to be done concerning their roles in chemoresistance, including overlap with lncPVT1 (46).

## Lung Cancer

Lung cancer remains the most prevalent cancer among both sexes (17). Non-small cell lung cancer (NSCLC) accounts for approximately 90% of lung cancers and the majority of patients are diagnosed at later stages (47). Cisplatin is a common first-line adjuvant chemotherapeutic for NSCLC, however many cancers are chemoresistant and have poor response to treatment (48–50).

LncPVT1 can promote cisplatin resistance in NSCLC by acting as a ceRNA for miR-216b and upregulating downstream Beclin-1 (51). Levels of lncPVT1 were significantly increased, while levels of miR-216b were reduced, in the A549/DDP cisplatin-resistant NSCLC cell line. PVT1 knockdown promoted apoptosis and suppressed autophagy. Beclin-1 plays a complex role in regulating both autophagy and apoptosis and it was found to be negatively associated with tumor recurrence rate in NSCLC (52, 53).

Other ncRNA have been shown to modulate chemoresistance by targeting Beclin-1. Mir-216b can modulate vemurafenib sensitivity in melanoma by targeting Beclin-1 and associated proteins (UVRAG and ATG5) (54). Negative regulation of Beclin-1 by miR-30d in anaplastic thyroid carcinoma (ATC) has been shown to suppress autophagy and promote apoptosis, and lower levels of miR-30d in ATC cells were correlated with cisplatin-resistance (55). The role of lncPVT1 in these contexts has not been studied.

LncPVT1 can behave as a ceRNA for at least four other miRNAs in NSCLC: miR-195, miR-199-5p, mir-424-5p, and miR-497 (56–60). Regulatory axes containing miR-195 and miR-424-5p are of particular interest since they have been shown to mediate radiosensitivity, a crucial component of multi-modal lung cancer treatment (56, 59). PVT1-derived miR-1204 has also been shown to promote cell proliferation in NSCLC by regulating paired-like homeodomain 1 (PITX1), a member of the RIEG/PITX family involved in organ development (61). LncPVT1 can also epigenetically downregulate large tumor suppressor kinase 2 (LATS2) in NSCLC via recruitment of EZH2 and methylation of the *LATS2* promoter, similar to the mechanism in cervical cancer (40). Further research is needed to uncover the molecular targets of other microRNA and potential mechanisms contributing toward chemoresistance in lung cancer.

## Osteosarcoma

Osteosarcoma is a rare sarcoma with global incidence of 3.4 cases per million, occurring mostly among younger age groups (62). Five-year survival has improved significantly since the introduction of chemotherapeutics, but high grade and metastatic patients still have dismal outcomes. Doxorubicin and cisplatin are ubiquitous components of osteosarcoma regimens, while gemcitabine is primarily used in patients with unresectable or recurrent disease (62, 63).

LncPVT1 can promote gemcitabine resistance in osteosarcoma by serving as a ceRNA of miR-152 and disinhibiting c-MET and the downstream PI3K/AKT pathway (64). PVT1 was upregulated and miR-152 was downregulated in the MG63/DOX chemoresistant osteosarcoma cell line. Overexpression of PVT1 attenuated gemcitabine-mediated inhibition of tumor growth. Previous studies have shown that c-MET mediates chemoresistance to cisplatin in osteosarcoma and that miR-152 acts as a tumor suppressor by targeting c-MET in oral squamous cell carcinoma (65, 66).

Alternatively, circRNA PVT1 may contribute to doxorubicin and cisplatin resistance in osteosarcoma by regulating multidrug resistance protein 1 (MDR1) (67). CircRNA PVT1 knockdown was shown to decrease levels of MDR1 and reverse doxorubicin and cisplatin resistance in chemoresistant cell lines. However, the specific mechanism of regulation was not clearly demonstrated. LncPVT1 can also alter glucose metabolism in osteosarcoma by acting as a ceRNA for miR-497 and disinhibiting hexokinase 2, consequently increasing glucose uptake and lactate production (68). It can also inhibit apoptosis, cell cycle arrest, and invasion/migration by acting as a ceRNA for of miR-195 and upregulating BCL2, CCND1, and FASN, respectively, in the U2OS cell line (69).

## Pancreatic Cancer

Pancreatic cancer (PC) is the fourth most common cancer in the United States (17). The typical adjuvant chemotherapeutic regimen generally consists of either gemcitabine or 5-flurouracil and leucovorin (70). Previous research indicated that PVT1 overexpression may induce gemcitabine resistance in PC (71). Meanwhile, overexpression of miR-1207-3p and miR-1207-5p inhibited proliferation and increased apoptosis after gemcitabine treatment. C-Src was identified as a potential target of miR-1207-5p and experiments confirmed that miR-1207-5p could reduce levels of c-Src in both PC cell lines and tissue. MiR-1207-3p was similarly shown to target RhoA, which has been shown to play a complex role in carcinogenesis (13, 72). Future research can hopefully elucidate the role of these pathways and their downstream targets in contributing to chemoresistance.

Additionally, gemcitabine treatment was shown to upregulate expression of miRNA processing enzymes, Drosha and DGCR8, leading to increased processing of lncPVT1 into mature miR-1207-5p/3p transcripts, thereby inhibiting downstream targets (13, 64). This unique regulatory relationship of differential processing of lncPVT1 has not been well-studied with respect to chemoresistance. Similar to its activity in cervical cancer, lncPVT1 can also upregulate the SMAD/TGF-β pathway and promote EMT in PC, although the mechanism by which this occurs has not yet been uncovered (73).

## Other Cancers

There is evidence that lncPVT1 and its associated miRNA transcripts can modulate chemoresistance in several other cancers, although their molecular targets have not been identified or their role in influencing response to chemotherapeutics has not yet been studied. It is critical to further study the role of these pathways in chemoresistance.

Gallbladder cancer (GBC) is rare in the United States, but prevalent among certain populations, such as North and South American Indians (74). GBC is overwhelmingly diagnosed at later stages and 5-year survival remains low (75). Cisplatin is a component of first-line therapy and resistance can impede treatment (76). MiR-1207-5p has been shown to decrease cisplatin sensitivity in GBC by an unknown mechanism (77). Levels of MiR-1207-5p correlated with both proliferative and apoptotic markers in this study, inconsistent with a uniform role in GBC. LncPVT1 can act as a ceRNA for miR-143 in GBC, disinhibiting hexokinase 2, and promoting cell proliferation invasion and migration (78, 79). Hexokinase 2 has also been shown to be regulated by miR-143 in other cancers, including colon, prostate, and breast (80). It is important to evaluate the extent these independent pathways may contribute to chemoresistance in GBC.

Hepatocellular carcinoma (HCC) is the fifth-most commonly diagnosed cancer globally, but accounts for a disproportionate amount of cancer-related deaths due to its difficult to treat nature (17, 81). Although surgical resection, radiofrequency ablation, and transplantation represent curative approaches for early stage cancers, there is a lack of systemic therapy available for more advanced cases (82). LncPVT1 has been shown to be upregulated in HCC tissues and associated with recurrence (83). LncPVT1 can serve as a ceRNA for miR-186-5P, thereby disinhibiting downstream yes-associated protein 1 (YAP1) to promote tumorigenesis in HCC (84). LncPVT1 can also recruit EZH2 to stabilize MDM2 and repress tumor suppressor p53 in HCC (42). MiR-424-5p is yet another proposed ceRNA target of PVT1, although further research are needed to demonstrate *in vitro* interaction (85).

Nasopharyngeal carcinoma (NPC) is a relatively rare cancer in the United States but has increased incidence in parts of Southern China (17, 86, 87). Typically, radiotherapy alone is used in treatment of early-stage NPC, but advanced disease usually necessitates the use of chemotherapy (88). MiR-1204 was shown to be downregulated in paclitaxel-resistant NPC cell lines, and restoration of miR-1204 was shown to resensitize NPC cells to paclitaxel *in vitro* and inhibit tumor growth *in vivo* (89). While there is evidence that miR-1204 can modulate paclitaxel-resistance in NPC, a molecular target has not yet been identified and the role of other ncRNA from the PVT1 locus has not been studied.

Ovarian cancer (OC) is the fifth leading cause of cancer-related death among women in the United States and chemoresistance plays a significant role in treatment failure in high-grade and recurrent subtypes (17, 90–92). MiR-1207 was upregulated in OC tissues and shown to target negative regulators of Wnt/β-catenin signaling pathway, including SFRP1, AXIN2, and ICAT, thereby promoting development of ovarian cancer stem cell-like traits (93). Previous studies have shown that genes involved in the Wnt signaling pathway are associated with chemoresistance in OC, highlighting the need for further research regarding the role of miR-1207 and related ncRNA in this context (94, 95).

Prostate cancer (PCa) is the second-most commonly diagnosed cancer among men (17, 81). Androgen-deprivation is the most common first-line therapy used in the treatment of PCa and the development of androgen-independent or castration-resistant PCa presents a significant obstacle to treatment (96, 97). MiR-1207-3p was found to be underexpressed in PCa cell lines and shown to target fibronectin type II domain containing 1 (FNDC1), thereby leading to downregulation of fibronectin 1 (FN1) and loss of androgen receptor expression (98). Future research should study the effects of miR-1207-3P in augmenting androgen-deprivation therapies. Overexpression of LncPVT1 was shown to be correlated with epigenetic silencing of miR-146a and increased cell survival in PCa (99). The mechanism by which this epigenetic regulation occurs was not identified and the influence on chemoresistance has not been studied.

Thyroid cancer (TC) is the ninth-most commonly diagnosed cancer worldwide, with a threefold higher incidence among women (17, 81). Several studies have implicated PVT1 in thyroid cancer and have shown it is significantly upregulated (100, 101). LncPVT1 acts as a ceRNA for miR-30b in papillary thyroid carcinoma (PTC), thereby disinhibiting IGFR1 and promoting cell proliferation, invasion, migration and EMT (102). LncPVT1 has also been shown to recruit EZH2 to reduce activity of the thyroid-stimulating hormone receptor (TSHR) in TC (41). These results are interesting although their significance in chemoresistance is not well-understood and warrants further study.

## Conclusion

**Figure 2:**
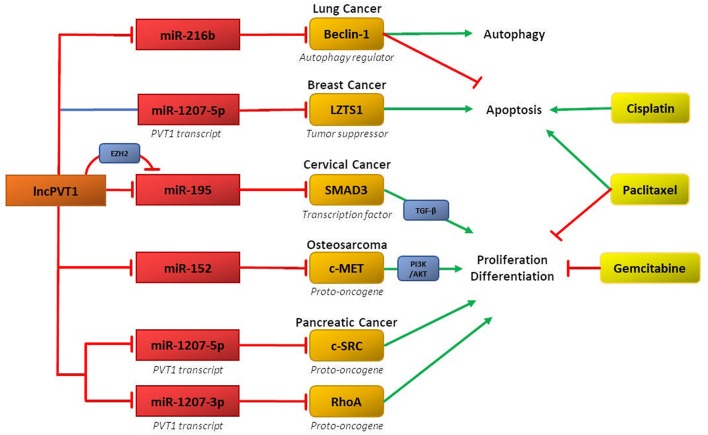
LncPVT1 and its associated microRNAs induce chemoresistance in various cancers by regulating oncogenes or tumor suppressor genes. Affected downstream pathways include the TGF-β and PI3K-AKTpathways. Also depicted is epigenetic inhibition of target genes via EZH2.

Recent research demonstrates that ceRNA networks involving lncPVT1 and its associated miRNAs can mediate chemoresistance in several cancers (Figure 2). The primary regulatory motif involves lncPVT1 acting as a ceRNA for specific miRNA and consequently disinhibiting downstream genes and proteins involved in promoting chemoresistance, such as anti-apoptotic proteins, cell cycle regulators, and mediators of EMT. Interestingly, differential processing of lncPVT1 in various cancers can also increase or decrease levels of PVT1-derived miRNAs, which can inhibit downstream targets. Alternatively, lncPVT1 or its associated transcripts can recruit epigenetic modifiers, such as EZH2, to modify expression of target genes.

In addition to the role of lncPVT1 in cisplatin resistance in gallbladder, lung, and osteosarcoma discussed here, lncPVT1 has also been shown to promote cisplatin resistance in gastric and colorectal cancers, although the molecular mechanisms and associated ceRNA networks have not been studied thoroughly and are promising for further research (103, 104). We also presented the first study implicating circRNA PVT1 in chemoresistance in osteosarcoma (67). It has previously been shown to have ceRNA activity in colorectal, gastric, and non-small cell lung cancers (105–107), although its chemoresistant role in other cancers has not been well-studied.

The majority of chemotherapeutics discussed here represent first-line therapies for their respective cancers, such as paclitaxel and cisplatin. However, often the most difficult to treat cases involved treatment with second- or third-line therapies, and thus future research should also consider the potential role of ncRNA to enhance the efficacy of these drugs.

The *PVT1* locus in particular is a promising area of study in terms of the discovery of new ceRNA networks in cancer. Further identification of regulatory networks could potentially introduce a new class of targeted therapeutics. Additional roles could include the co-delivery of drugs to potentiate existing therapeutics, especially difficult to treat chemoresistant cancers involving second- or -third line treatments.

## Author Contributions

AK wrote the first draft of the manuscript. OO reviewed, edited, and approved final version of the manuscript.

### Conflict of Interest Statement

OO is a co-founder of NucleoBio, Inc., a City University of New York start-up biotechnology company. The remaining author declares that the research was conducted in the absence of any commercial or financial relationships that could be construed as a potential conflict of interest.
